# GLP-1 Inhibits High-Glucose-Induced Oxidative Injury of Vascular Endothelial Cells

**DOI:** 10.1038/s41598-017-06712-z

**Published:** 2017-08-14

**Authors:** Quan Li, Yajun Lin, Shu Wang, Lina Zhang, Lixin Guo

**Affiliations:** 1 Graduate School of Peking Union Medical College and Chinese Academy of Medical Sciences, Beijing, China; 2 Department of Endocrinology, Ministry of Health, Beijing Hospital, Beijing, China; 3 The Key Laboratory of Geriatrics, Beijing Hospital & Beijing Institute of Geriatrics, Ministry of Health, Beijing, China

## Abstract

The aim of this work was to evaluate the effects of glucagon-like peptide-1 (GLP-1) on high-glucose-induced oxidative stress and investigate the possible mechanisms underlying this process. We measured reactive oxygen species (ROS) production, cell apoptosis, the expression of NOX4 and its subunits, and p47phox translocation in human umbilical vein endothelial cells (HUVECs). An experimental type 2 diabetes model was induced using streptozotocin in male Sprague-Dawley rats. Fasting blood glucose (FBG), fasting insulin (FINS), total cholesterol (TC), triglycerides (TGs), and free fatty acid (FFA) were measured. Histomorphological analysis of the aorta was performed using hematoxylin-eosin staining. NOX4 and VCAM-1 expression in the aorta was measured. We found that high-glucose-induced ROS production and apoptosis were inhibited by GLP-1 treatment. High glucose caused upregulation of NOX4, p47phox, and Rac-1 and translocation of p47phox but had no effect on the cells pretreated with GLP-1. Furthermore, in the diabetic group, FBG, FINS, TG, TC, and FFA were increased, and NOX4 and VCAM-1 levels were also elevated. However, GLP-1 attenuated all these changes. GLP-1 ameliorated high-glucose-induced oxidative stress by inhibiting NOX4, p47phox, and Rac-1 expression and translocation of p47phox, suggesting its clinical usefulness in diabetic vascular complications.

## Introduction

Cardiovascular complications are major problems associated with diabetes, leading to numerous hospital readmissions and high mortality and morbidity. Endothelial damage is considered to be an early indicator of diabetic macrovascular complications. A number of studies have shown that high glucose was crucially involved in endothelial damage through the overproduction of reactive oxygen species (ROS)^1–4^. Approximately 60% of total vascular superoxide was derived from NADPH oxidase (NOX) in diseased human coronary arteries^5^, and NOX4 was the major source of ROS^6^ in human umbilical vein endothelial cells(HUVECs). Therefore, a therapeutic approach that prevents high-glucose-induced oxidative stress may reduce the risk of diabetic complications.

Glucagon-like peptide-1(GLP-1), which is synthesized and secreted by intestinal L-cells, was identified as a protective target for type 2 diabetes. Furthermore, several studies have shown the beneficial effects of GLP-1 on cardiovascular function^7–10^. GLP-1 reduced ischemic damage and decreased myocardial infarct size in experimental myocardial infarction in mice^11, 12^, and recent data indicated that GLP-1 administration improved endothelial function in diabetic patients. In addition, several studies have demonstrated that GLP-1 suppressed oxidative stress in endothelial cells and diabetic rats^13, 14^. GLP-1 was capable of exerting direct protective effects against oxidative stress in the aorta of diabetic mice^15^. Thus, we aimed to elucidate the protective effects of GLP-1 on high-glucose-induced oxidative stress *in vivo* and *in vitro* and to investigate the mechanism of this process.

## Results

### Exenatide reduced oxidative damage to the aorta of type 2 diabetic (DM) rats

**Figure 1:**
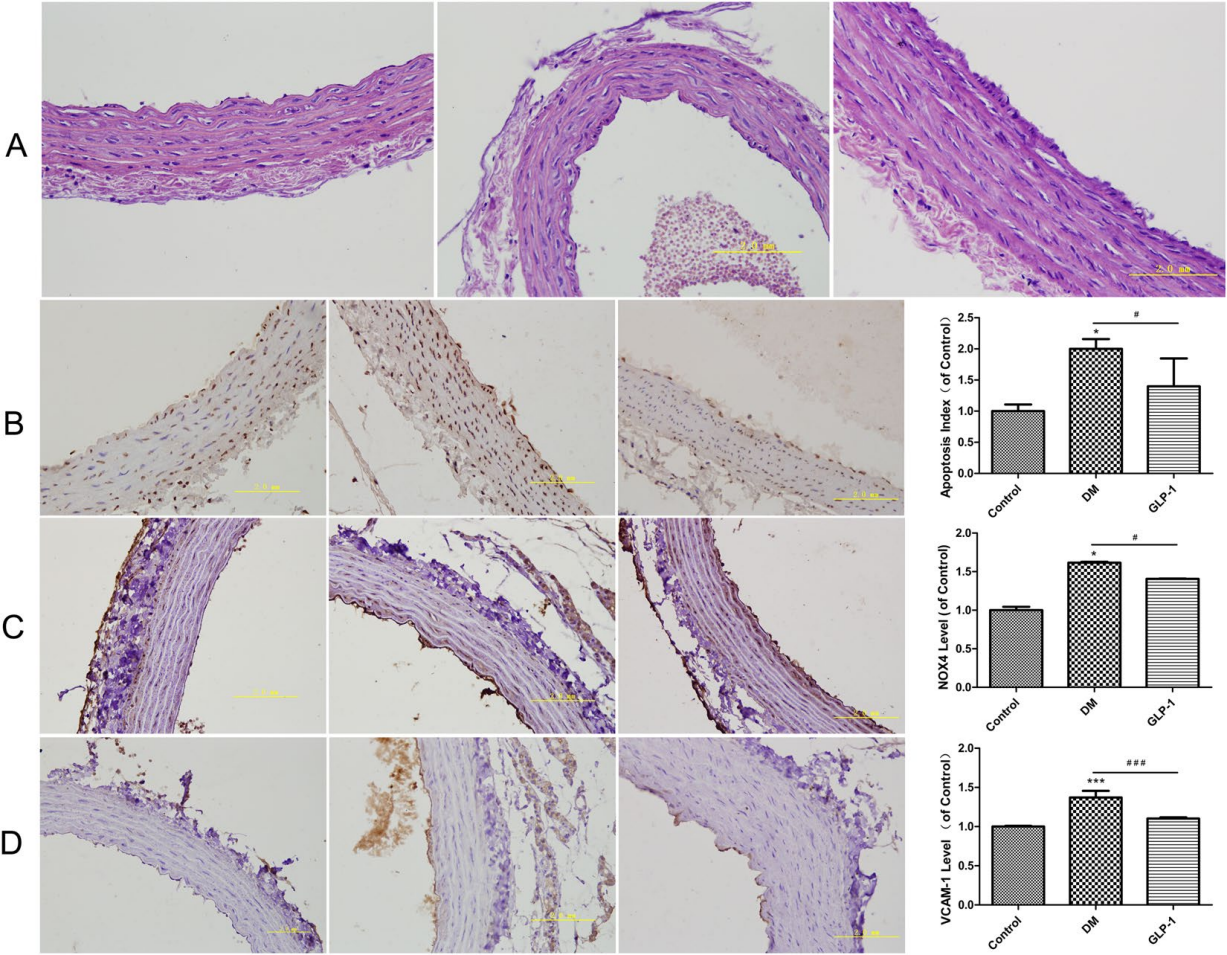
HE staining of aortic morphology, apoptosis, NOX4 and VCAM-1 level in rat aorta. HE staining of aortic morphology (**A**). TUNEL assay measuring the apoptosis of aortic endothelial cells (**B**). The levels of NOX4 (**C**). The levels of VCAM-1 in rat aorta (**D**). Data are shown as the mean ± SEM, n = 5 independent experiments. *P < 0.05 *vs* control. ^#^ *P* < 0.05 *vs* DM group, ***P < 0.001 *vs* control. ^###^ *P* < 0.001 *vs* DM group.

Fasting blood glucose (FBG), fasting insulin (FINS), triglyceride (TG), total cholesterol (TC), and free fatty acid (FFA) levels were significantly increased in DM rats; however, these increases were prevented by exenatide (Table 1). Rats in the diabetic group had the locally thickened intima and tunica elastica, rough intima, endothelial cell protrusions, irregularly shaped endothelial cells, and disordered arrangement of the smooth muscle cells in the aorta. The aorta intima was slightly thickened and rough, the endothelial cells and smooth muscle cells were orderly and the tunica elastica was lightly thickened in the diabetic group treated with exenatide compared to the characteristics of the diabetic group (Fig. 1A). The NOX4 and VCAM-1 levels in the arteries of DM rats were markedly elevated and accompanied by increased endothelial cell apoptosis. However, exenatide reversed these changes (Fig. 1B, D).

### GLP-1 inhibited the decreased HUVEC viability induced by high glucose

**Figure 2:**
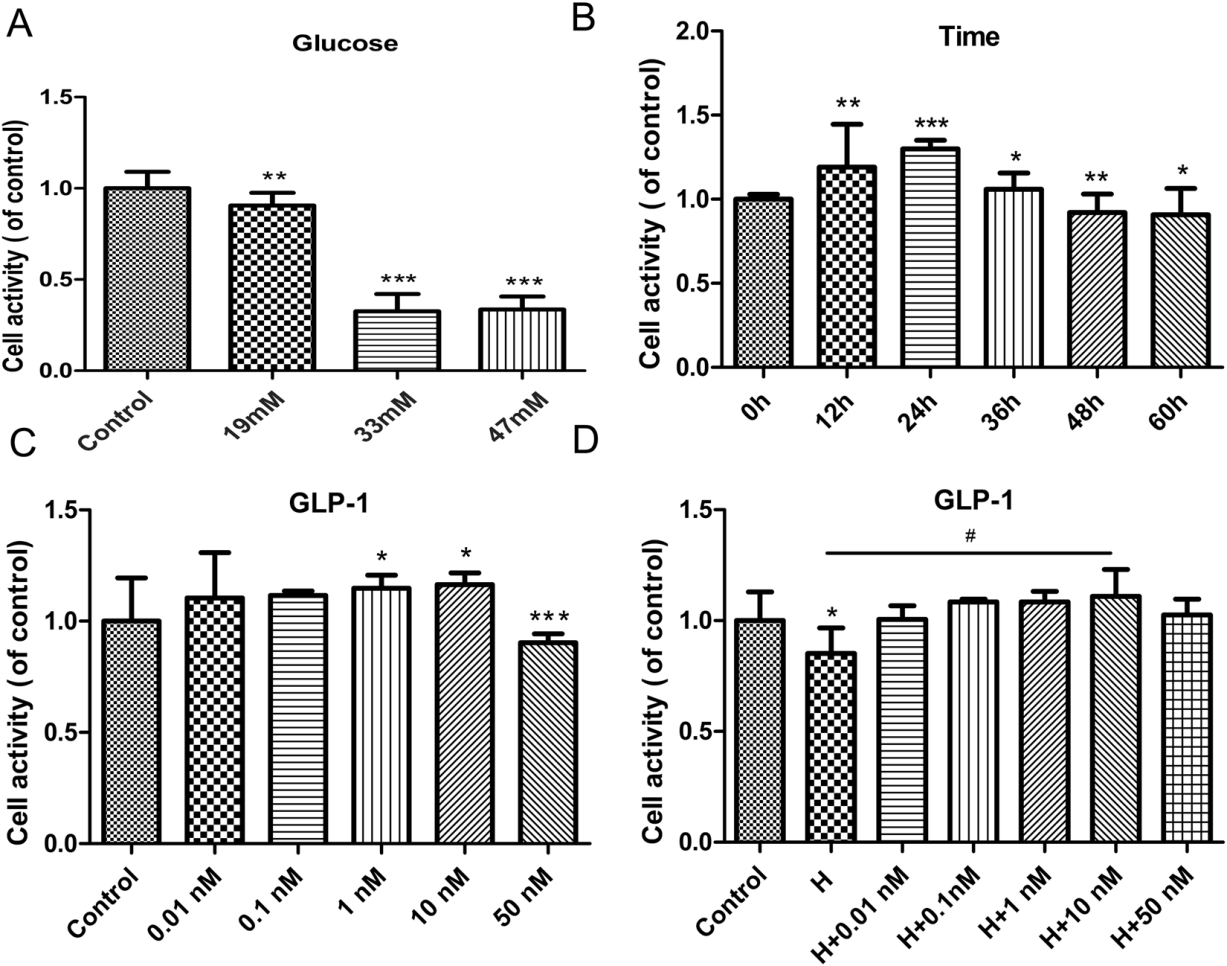
The changes in cell viability of HUVECs. (**A**) The cell viability of HUVECs treated with different concentration of glucose for 48 h (**B**). The cell viability of HUVECs treated with 33 mmol/L glucose for various amounts of time (**C**). Cell viability in HUVECs treated with different concentration of GLP-1 for 48 h (**D**). Cell viability in HUVECs treated with different concentration of GLP-1 and 33 mmol/L glucosefor 48 h. Data are shown as the mean ± SEM, n = 5 independent experiments. *P < 0.05 *vs* control, ^#^ *P* < 0.05 *vs* high glucose, **P < 0.01 *vs* control, ***P < 0.001 *vs* control.

First, we determined the concentration of glucose that induced HUVEC injury. As shown in Fig. 2A, a high concentration of glucose decreased cell viability, and glucose had similar effects at concentrations of 33 and 47 mmol/L. Next, we evaluated the viability of cells treated with 33 mmol/L glucose at multiple time points (Fig. 2B). Cell viability reached the lowest value when HUVECs were exposed to glucose for 48 h. In additional experiments, HUVECs were incubated with different concentrations of GLP-1(0.01, 0.1, 1.0, 10, 50 nmol/L) in the presence or absence of 33 mmol/L glucose for 48 h. Compared with control cells, GLP-1 significantly increased cell viability in HUVECs pretreated with 10 nmol/L GLP-1. However, 50 nmol/L GLP-1 decreased cell viability (Fig. 2C). These results demonstrated that GLP-1 inhibits decreased cell viability induced by glucose, and all subsequent experiments were performed with 33 mmol/L glucose and 10 nmol/L GLP-1.

### GLP-1 attenuated apoptosis induced by high glucose and ROS generation

**Figure 3:**
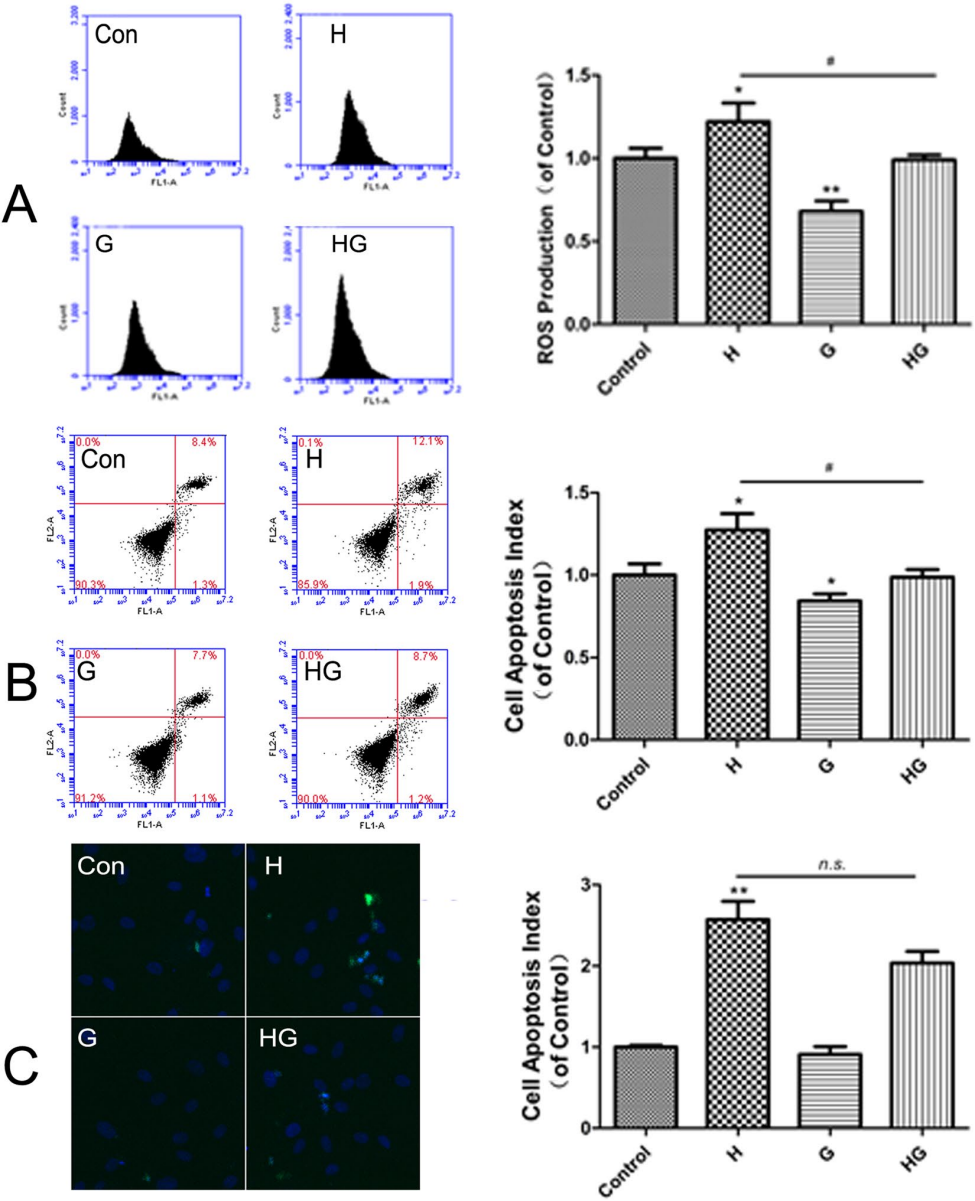
GLP-1 pretreatment attenuated ROS generation and apoptosis in HUVECs induced by 33 mmol/L glucose for 48 h. GLP-1 decreased ROS production in HUVECs induced by high glucose (**A**). GLP-1 decreased the apoptosis rate in HUVECs induced by high glucose, as detected using an Annexin V-PI assay (**B**). GLP-1 decreased the apoptosis rate in HUVECs induced by high glucose, as detected by TUNEL (**C**). Data are shown as the mean ± SEM, n = 5 independent experiments. *P < 0.05 *vs* control. ^#^ *P* < 0.05 *vs* high glucose, **P < 0.01 *vs* control. ^n.s.^ *P* > 0.05 *vs* high glucose.

As shown in Fig. 3A, HUVECs treated with 33 mmol/L glucose for 48 h showed a significant increase in ROS detected by 2′,7′-dichlorofluorescein diacetate (DCFH-DA) fluorescence and flow cytometry. However, the changes were partially reversed by GLP-1. In addition, the proportion of apoptotic cells was quantified by an Annexin-FITC/PI assay (Fig. 3B). After the cells were incubated with 33 mmol/L glucose for 48 h, the percentages of early and late apoptotic cells were both increased. This increment was attenuated when the cells were pretreated with GLP-1. The GLP-1 + 33 mmol glucose group had slightly reduced cell apoptosis compared with that in the high glucose group as shown by terminal deoxynucleotidyltransferase-mediated dUTP nick end labeling(TUNEL) assays, but this difference was not significant (Fig. 3C).

### High glucose induced ROS production by activating NOX4

**Figure 4:**
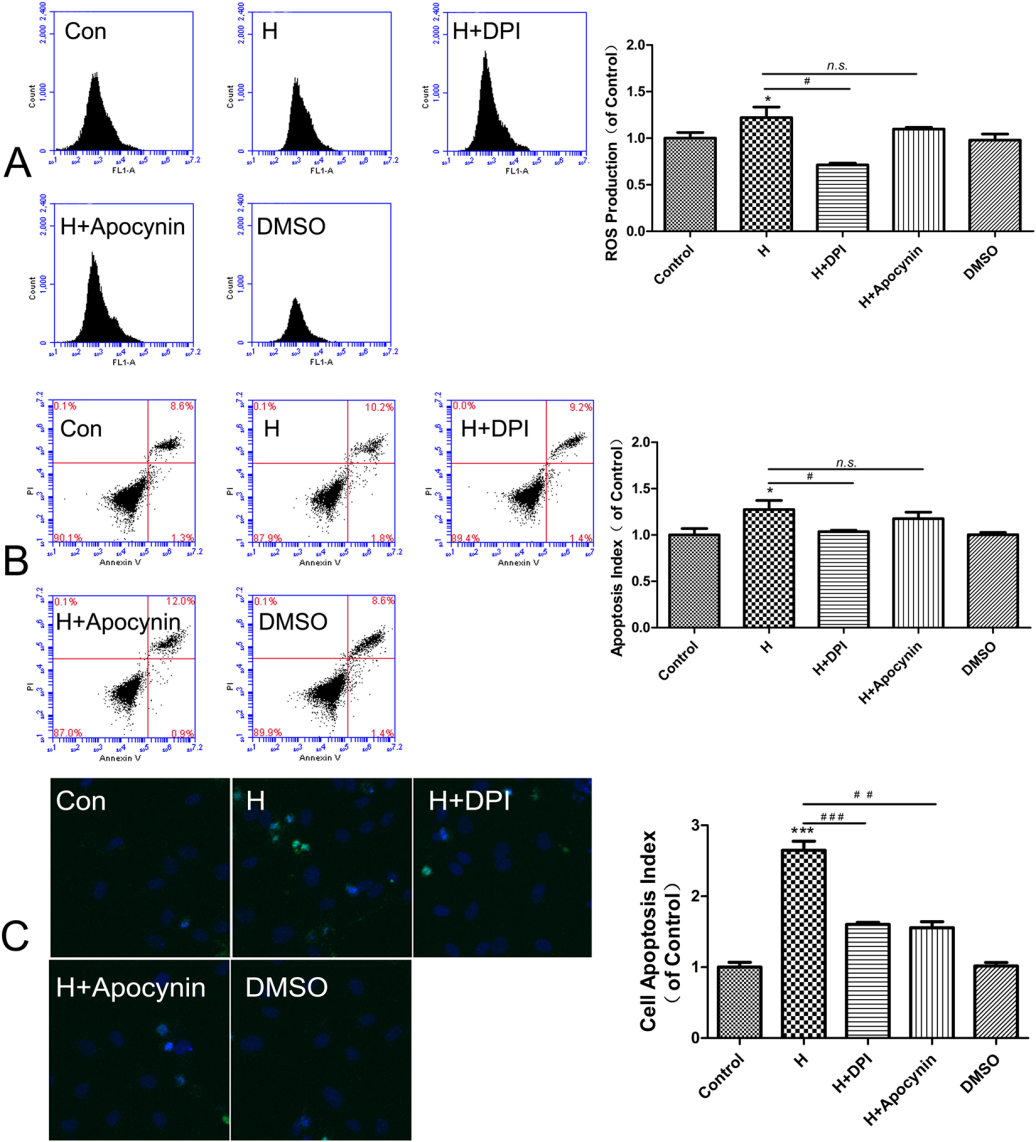
DPI and Apocynin pretreatment attenuated ROS generation and apoptosis in HUVECs induced by 33 mmol/L glucose for 48 h. DPI and Apocynin decreased ROS production in HUVECs induced by high glucose (**A**). DPI and Apocynin decreased the apoptosis rate in HUVECs induced by high glucose, as detected using an Annexin V-PI assay (**B**). DPI and Apocynin decreased the apoptosis rate in HUVECs induced by high glucose, as detected by TUNEL (**C**). Data are shown as the mean ± SEM, n = 5 independent experiments. *P < 0.05 vs control, ^#^P < 0.05 vs high glucose, **P < 0.01 vs control, ***P < 0.001 vs control, ^###^P < 0.001 vs high glucose, ^n.s.^P > 0.05 vs high glucose.

Our observations indicated that ROS production was significantly elevated in HUVECs incubated with high glucose. NOX4 is a leading candidate for ROS generation in endothelial cells. To determine the contribution of NOX to high-glucose-induced oxidative stress of HUVECs, we investigated the effects of the NOX inhibitor DPI (2.5 μmol/L) and the p47phox translocation inhibitor Apocynin(1 μmol/L). As shown in Fig. 4A, DPI significantly inhibited the generation of ROS in response to high glucose treatment. High-glucose-induced HUVEC apoptosis was attenuated by pretreatment of DPI as shown by an Annexin-FITC/PI assay (Fig. 4B).TUNEL assays also indicated that HUVEC apoptosis induced by high glucose was attenuated by pretreatment with DPI and Apocynin (Fig. 4C).

### The effect of GLP-1 on NOX4, p47phox, and Rac-1 expression and p47phox translocation

**Figure 5:**
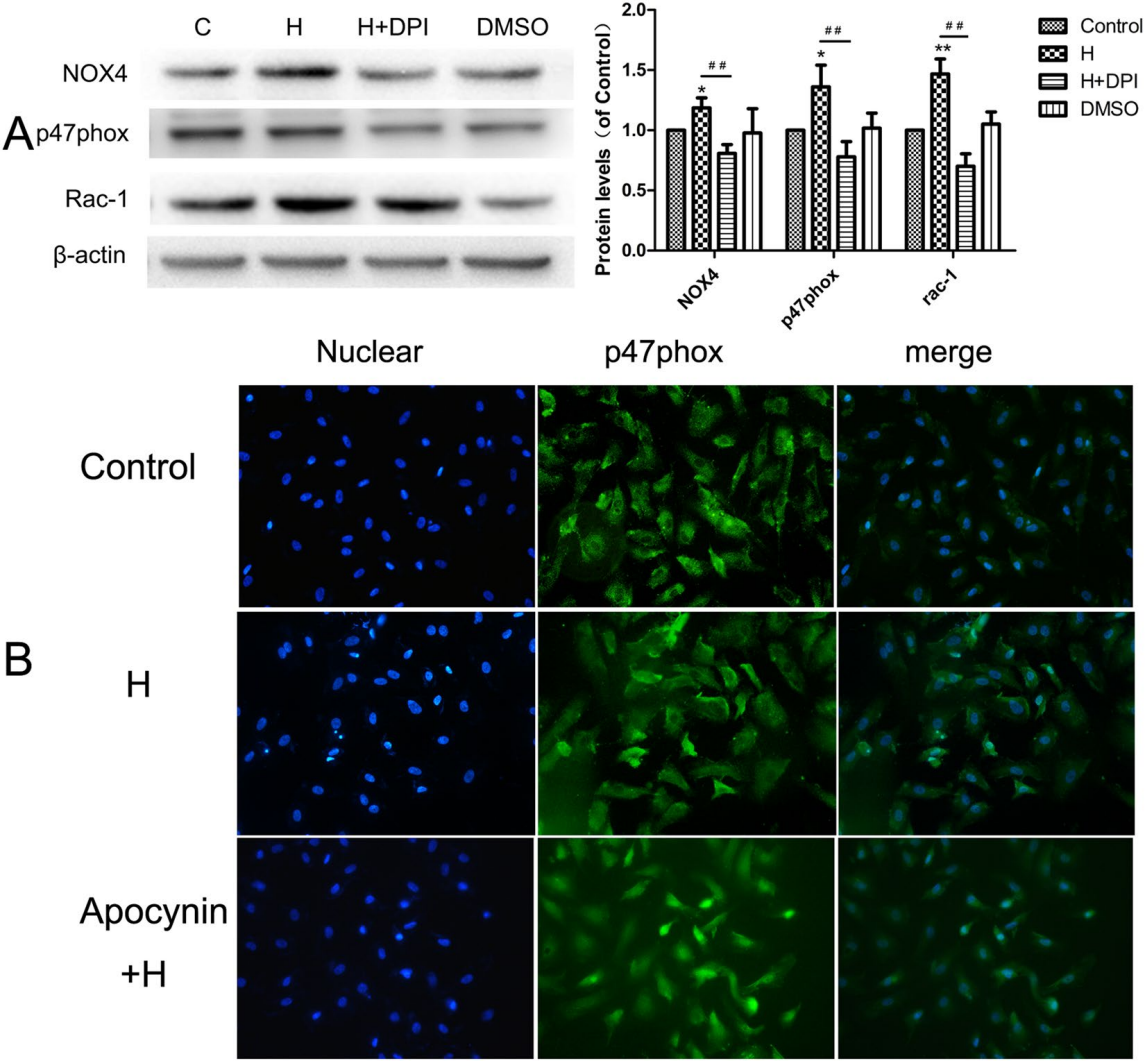
DPI and Apovynin pretreatment attenuated NOX4, p47phox and Rac-1 expression and p47phox translocation. DPI decreased NOX4, p47phox and Rac-1 expression (**A**). Apocynin decreased p47phox translocation induced by high glucose (**B**). Data are shown as the mean ± SEM, n = 5 independent experiments. *P < 0.05 *vs* control, **P < 0.01 *vs* control, ^##^ *P* < 0.01 *vs* high glucose.

To elucidate the molecular mechanisms involved in glucose-induced oxidative stress, we investigated the protein expression of NOX4 and its subunits. As shown in Fig. 5A, the expression of NOX4, p47phox, and Rac-1 in HUVECs treated by 33 mmol/L glucose for 48 h was elevated, and pretreatment with DPI decreased the expression of NOX4, p47phox, and Rac-1 compared to that of treatment with glucose alone. The translocation of p47phox into the cell membrane compartment is an indicator of NOX activation. We assayed the immunofluorescence of the p47phox protein to further explore NOX activation in response to high glucose. As shown in Fig. 5B, the control group exhibited faint p47phox fluorescence (green) that was diffusely distributed within the cell. In the glucose group, p47phox translocated to the membrane, but translocation of p47phox was reversed following Apocynin treatment. These results indicate that the suppression of NOX4 can decrease ROS generation induced by high glucose.

### GLP-1 inhibited the glucose-induced activation of NOX4

**Figure 6:**
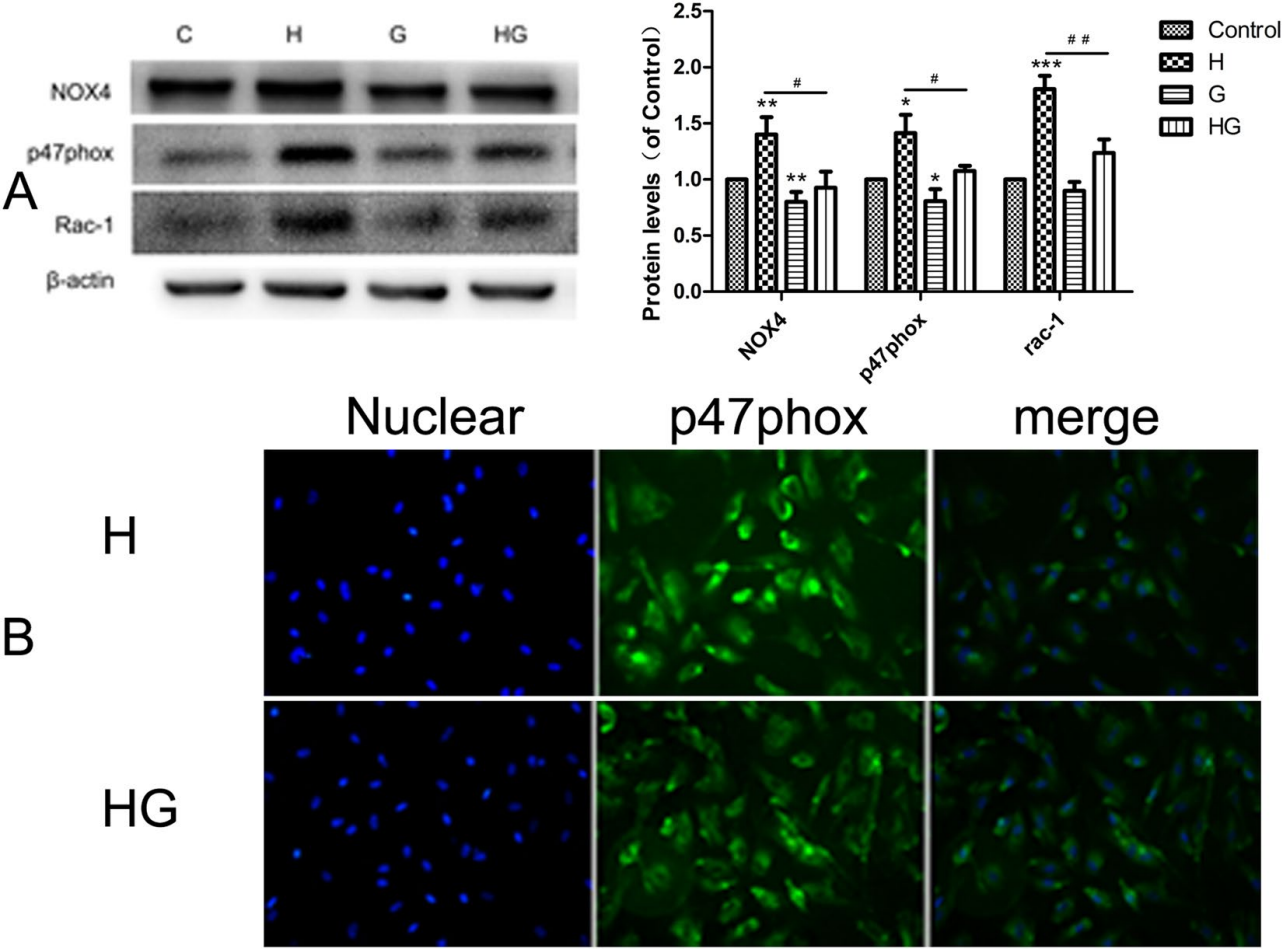
GLP-1 pretreatment attenuated NOX4, p47phox and Rac-1 expression and p47phox translocation. GLP-1 decreased NOX4, p47phox and Rac-1 expression (**A**). GLP-1 decreased p47phox translocation induced by high glucose (**B**). Data are shown as the mean ± SEM, n = 3 independent experiments. *P < 0.05 *vs* control. ^#^ *P* < 0.05 *vs* high glucose, **P < 0.01 *vs* control, ^##^ *P* < 0.01 *vs* high glucose, ***P < 0.001 *vs* control.

As shown in Fig. 6, NOX4, p47phox, and Rac-1 were upregulated after stimulation with high glucose, accompanied by p47phox translocation to the membrane. Pretreatment with 10 nmol/L GLP-1 prevented the upregulation of NOX4, p47phox and Rac-1 and attenuated the translocation of p47phox. These results revealed that GLP-1 suppressed NOX4 activation, leading to reduced oxidative stress.

## Discussion

This study showed that GLP-1 could protect macrovascular endothelial cells against oxidative stress both in HUVECs and in diabetic rats. Previously, researchers have reported the protective effects of GLP-1 on oxidative stress. However, hyperglycemia is a distinctive characteristic of diabetes, and few studies have assessed the effects of GLP-1 on high-glucose-induced oxidative stress. Therefore, we investigated this using HUVECs and diabetic rats. HUVECs are a classic model of atherosclerosis *in vitro*. NOX4 is a leading candidate for ROS generation in endothelial cells. In our study, we found that the protective effects of GLP-1 are dependent on inhibition of NOX4. These findings indicated that GLP-1 might be beneficial in delaying endothelial defects in diabetic vessels and contributing to reduced risk of diabetic cardiovascular complications.

Endothelial dysfunction induced by hyperglycemia plays an important role in the onset and progression of diabetic vascular complications. High glucose concentrations can trigger ROS generation and cell apoptosis, induce inflammation, and finally promote pathogenesis of diabetes-accelerated atherosclerosis. In accordance with most studies, we found that after the treatment of HUVECs with high glucose, as well as in aorta endothelium of diabetic rats, cell viability was increased, and the apoptosis index and VCAM-1 levels were elevated. Therefore, increased production of superoxide via NOX may lead to the enhanced expression of adhesion molecules in diabetic vasculature^16^. Consistent with these previous results, we showed that VCAM-1 levels were increased in the aorta of streptozotocin-treated rats.

The overproduction of ROS plays a pivotal role in the development of diabetic vascular complications and is often associated with endothelial dysfunction^17^. We next sought to investigate the mechanisms of increased oxidative stress induced by high glucose. ROS are produced through the mitochondrial electron transfer chain, NADPH oxidases, NOS, peroxidases, and other enzymes. NOX4 is the major enzyme in the vasculature that interferes with endothelial dysfunction^18, 19^. The NOX enzyme complex is composed of a membrane-associated subunit (gp91phox and p22phox) and cytosolic subunits (p40phox, p47phox, p67phox, and Rac-1). When activated, the cytosolic components translocate to the membrane, where they form a molecular cluster of the catalytically active oxidase. P47phox is a key protein in the assembly of NOX, leading to superoxide generation. We found that high glucose-induced ROS production in HUVECs was significantly reduced by DPI; high glucose-induced cell apoptosis in HUVECs was significantly reduced by DPI and Apocynin, suggesting that NOX4 and p47phox translocation play major rolesin ROS generation and cell apoptosis induced by hyperglycemia. The different results between Annexin-FITC/PI assays and TUNEL assays maybe due to differences in test sensitivity.

In our study, 10 nmol/L GLP-1 increased cell viability; however, 50 nmol/L GLP-1 decreased cell viability. Thus,10 nmol/L GLP-1 was used in the following experiments. The present study also demonstrated that 10 nmol/L GLP-1 pretreatment for 30 min could inhibit ROS generation and apoptosis in HUVECs exposed to high glucose by inhibiting NOX activity, which subsequently protected cells against oxidative stress-induced injuries. Previous studies have shown the beneficial effects of GLP-1 on oxidative stress-related impairment. Ishibashi Y. reported that GLP-1 exerted protective effects on advanced glycation end product-induced oxidative stress. Exendin-4 has been shown to alleviate angiotensin II-induced senescence in vascular smooth muscle cells and ameliorate peripheral vascular disease^20–22^. Furthermore, GLP-1 decreased high-glucose-induced levels of the NOX subunits, such as p47phox, gp91phox and p22phox, in cardiac microvascular endothelial cells^14^ and the cardiomyocytes of neonatal rats^23, 24^. We showed that hyperglycemia mediated the upregulation of NOX4, p47phox and Rac-1 protein levels, which were significantly reduced by GLP-1. Moreover, translocation of p47phox to the membrane was inhibited after GLP-1 treatment. Similarly, the overexpression of NOX4 and VCAM-1 in the streptozotocin-induced diabetic rat aorta was reversed by exenatide.

## Materials and Methods

### Reagents and antibodies

D-glucose, GLP-1(7–36 amides), DCFH-DA, DPI, and Apocynin were purchased from Sigma (St. Louis, MO, USA). Antibodies against NOX4, p47phox, and Rac-1 were acquired from Abcam. An antibody against VCAM-1 was from Santa Cruz Biotechnology, Inc. (Delaware, CA, USA). Secondary antibodies against rabbit or mouse IgG were obtained from Cell Signaling Technology(Danvers, MA, USA). Western Blot Luminol Reagent and PVDF membranes were purchased from Millipore (Billerica, MA, USA). The MTS assay kit was purchased from Promega (Madison, WI, USA). Annexin V-FITC/PI Apoptosis Detection Kits were obtained from Baosai Company (Beijing, China). The TUNEL assay kit was acquired from Roche (Mannheim, Germany). M199 culture medium and fetal bovine serum (FBS) were obtained from GibcoBRL (Grand Island, NY, USA).

### Cell culture

Human umbilical cords were collected after written informed consent was obtained from all study subject participants with healthy full-term and naturally delivered newborns at Beijing Hospital (China).The local human Investigation Ethics Committee at Beijing Hospital approved this study. All methods in this study were performed in accordance with the relevant guidelines and regulations. HUVECs were isolated from fresh umbilical veins as described by Jaffe *et al*.^15^, and the isolated cells were cultured in M199 medium supplemented with 20% fetal bovine serum, 2 mM glutamine, and antibiotics (100 U/ml penicillin G and 100 μg/ml streptomycin) at 37 °C in a humidified 5% CO_2_ atmosphere. HUVECs at passages 2–4 were used for this study. After reaching confluence, cells were incubated in low-serum medium (M199 containing the above except for 2% FBS) for 18-24 h before proceeding with further experiments.

### Cell viability assay

Cell viability was measured using the MTS kit according to the manufacturer’s instructions. HUVECs (3000 cells/well) were plated onto 96-well plates. All assays were performed in triplicate. After treatment, 10μl/ml MTS was added to the cell culture for 2 h at 37 °C in the dark, and the absorbance was measured at 490 nm using a microplate reader (SpectraMax 190, Sparta, WI, USA). Cell viability is expressed as a percentage of the control.

### Determination of apoptosis

#### AnnexinV-PI staining

For analysis of apoptosis in HUVECs, the cells were washed and double-stained with FITC-conjugated Annexin V and PI using the Annexin V and PI kit (Baosai, Beijing, China) according to the manufacturer’s protocol. The stained nuclei were immediately enumerated by FACS.

#### TUNEL staining

Nuclear fragmentation was detected by TUNEL staining with an apoptosis detection kit according to the manufacturer’s instructions or by incubating fixed cells (4% paraformaldehyde/PBS) with DAPI. Then, 500-700 cells in 10 randomly chosen fields from each dish were counted to assess the percentage of apoptotic nuclei, and at least 3 experiments were performed for each manipulation.

### Determination of ROS

Cells (3 × 10^5^ cells/ml) were incubated with the fluorescent probe DCFH-DA at a final concentration of 10 μM for 30 min at 37 °C in the dark. The cells were washed three times with PBS, trypsinized, and re-suspended, and then, 2′,7′-dichlorofluorescein fluorescence was measured by FACS.

### Western blot analysis

Western blots were employed to detect the levels of NOX4, p47phox and Rac-1. Equivalent amounts of protein from each sample were prepared and separated by SDS-PAGE (12% gels) followed by electrotransfer to PVDF membranes. Membranes were then incubated with blocking solution (Tris-buffered saline, 8% nonfat dry milk) for 2 h, followed by incubation with specific antibodies at 4 °C overnight. After extensive washes in TBST (containing 0.5% Tween-20 in TBS), the membranes were then incubated with horseradish peroxidase-conjugated secondary antibodies in TBST for 1 h. After further washes in TBST, the bands were detected with chemiluminescence detection agents. Blot densitometry was performed, and the bands were analyzed with ImageJ software.

### Immunohistochemistry analysis

For histological analysis, aorta tissues fixed with4% buffered paraformaldehyde were embedded in paraffin, and 4-μmthicksections were prepared. The sections were then stained with hematoxylin-eosin (HE). Immunohistochemical analyses were performed using antibodies against NOX4 and VCAM-1. The sections were deparaffinized and quenched in 3% H_2_O_2_ for 15 min to block endogenous peroxidase and then washed in PBS. The sections were subsequently incubated with anti-NOX4 or anti-VCAM-1 antibodies for 2 h, followed by incubation with a biotinylated secondary antibody and ABC reagent (Biomed Company, Beijing, China) as recommended by the manufacturer’s instructions. The color was developed by incubating the sections with diaminobenzidine as a substrate. The slides were counterstained with Mayer’s hematoxylin, and slides that were preincubated with BSA served as negative controls. Brownish yellow granular or linear deposits were interpreted as positive areas. All images were captured using an Olympus microscope (BX60, Olympus, Ina, Japan), and the mean optical density of NOX4 and VCAM-1 was analyzed with Image-Pro Plus 6.0 (Media Cybernetics).

### Animal study

Six-week-old male SD rats were purchased from the Peking University Health Science Center and used in the study. All procedures conducted with animals were approved by our institutional review board (Animal Experiments Ethics Board, Beijing Hospital, Beijing, China) and were carried out in accordance with the approved guidelines from the Institutional Animal Care and Use Committee of Beijing Hospital and conformed to the Guidelines for Proper Conduct of Animal Experiments of the Science Council of China. Thirty male SD rats were randomly divided into a normal control group (control group, n = 8) and diabetic model group (group DM, n = 22).The type 2 diabetic model was established by intraperitoneal injection of streptozotocin (30 mg/kg body wt; Sigma, St. Louis, MO) and orally fed a high-fat diet (20 g/day/rat) for a period of 28 days. Blood glucose was measured using Accusoft glucose test strips read on a glucometer (Roche Diagnostics, Laval, Quebec). Rats with fasting blood glucose levels ≥ 11.1 mM at 72 h after streptozotocin injection were considered to be diabetic. Eighteen diabetic rats induced successfully were randomly divided into diabetic (group DM, n = 9) and diabetic treated with exenatide (group GLP-1, n = 9) groups for 6 weeks. Rats in group GLP-1 were injected subcutaneously with exenatide (5μg/kg, twice a day), and rats in the normal control group and diabetic group were given an equivalent volume of normal saline by subcutaneous injection. The rats were euthanized at termination, a blood sample was withdrawn from the inferior vena cava, and aliquots were stored at -80 °C until use.

#### Measurement of plasma insulin and lipid levels

FBG, FINS, TG, TC, and FFA were measured. TG, TC and FFA were determined using an automatic analyzer (Roche, Basel, Switzerland). Serum insulin levels were measured by enzyme-linked immunosorbent assay (Jiancheng, Nanjing, China). The aorta was separated and fixed by formalin, and HE staining was performed to observe the histomorphology. The expression and distribution of NOX4 and VCAM-1 in aorta were detected by immunohistochemical staining. Cell apoptosis were detected by TUNEL assays.

#### Immunofluorescence of p47phox

HUVECs were seeded onto sterilized coverslips placed in a 6-well tissue culture plate. After treatment, the cells were fixed for 15 min in 4% paraformaldehyde/PBS at room temperature and then blocked with 3% BSA at RT for 15 min. Anti-p47phox antibody in PBS at a 1:50 dilution was added to each well for 1 h at 37 °C. After a wash, an FITC-conjugated antibody was added at a 1:50 dilution and incubated for 0.5 h at 37 °C. The coverslips were mounted on clean glass slides with mounting media, and DAPI was used to stain the nuclei. P47phox was imaged usinga fluorescence microscope (BX60, Olympus, Ina, Japan). P47phox was observed as green fluorescence and the nucleus as blue fluorescence.

**Table 1:** Plasma glucose, Plasma insulin, Total cholesterol, triglyceride, and Free fatty acids.

	Control	DM	GLP-1
Plasma glucose (mmol·L-1)	4.42 ± 0.69	19.89 ± 0.97^**^	15.30 ± 0.93^**,#^
Plasma insulin (kIU·L-1)	13.92 ± 2.23	28.52 ± 2.54^**^	22.36 ± 2.78^**^
Total cholesterol (mmol·L-1)	1.60 ± 0.35	2.70 ± 0.25^**^	2.02 ± 0.77^#^
Triglyceride (mmol·L-1)	0.97 ± 0.15	1.73 ± 0.24^*^	1.24 ± 0.32^##^
Free fatty acids (mmol·L-1)	1.45 ± 0.19	2.59 ± 0.43^**^	1.58 ± 0.36^##^

Data are shown as the mean ± SEM, n = 8 independent experiments. ^*^ *P* < 0.05 *vs* control, ^**^ *P* < 0.01 *vs* control. ^#^ *P* < 0.05 *vs* DM group, ^##^ *P* < 0.01 *vs* DM group.

### Statistical analysis

Data are expressed as the mean ± the SD. Statistical analysis was performed by Student’s test or one-way ANOVA followed by an LSD post-test. P < 0.05 was considered statistically significant.
